# Supplementary figures and images for: Heatwave-like events affect drone production and brood-care behaviour in bumblebees
Source: PeerJ. 2024 Mar 22;12:e17135. doi: 10.7717/peerj.17135 (PMC10962346; doi:10.7717/peerj.17135)

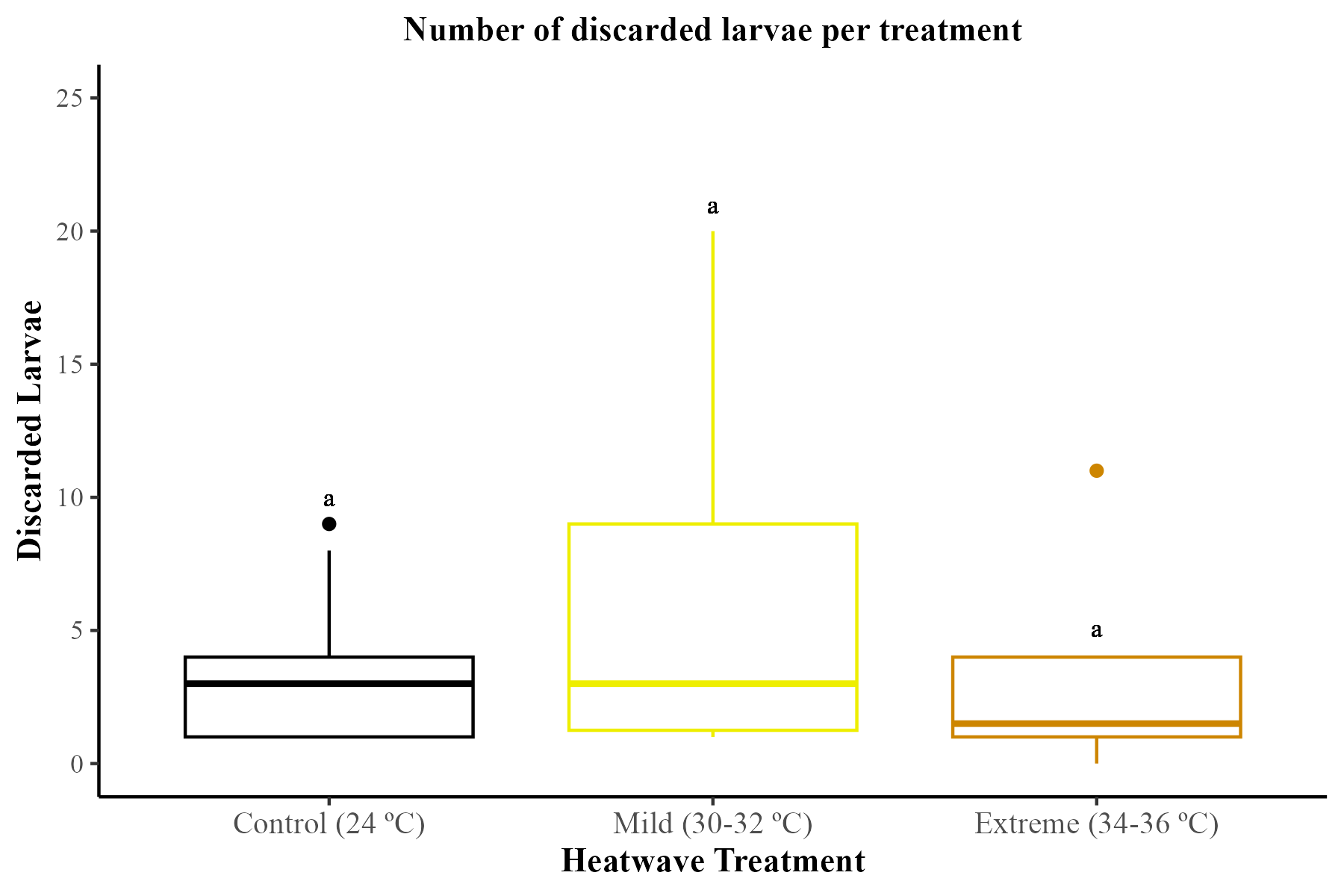

Supplement: Supplemental Information 1 — Production of larvae was assessed across 29 bumblebee microcolonies (Bombus terrestris audax) (Control = 9 microcolonies; mild heatwave = 10 microcolonies; extreme heatwave = 10 microcolonies). The boxes represent the interquartile ranges and the horizontal lines within the boxes show the medians. Whiskers extend to 1.5 times the interquartile range from the first and third quartiles, displaying the range of the data. Any data points outside these whiskers are considered outliers and are represented as individual points. [file peerj-12-17135-s001.png]

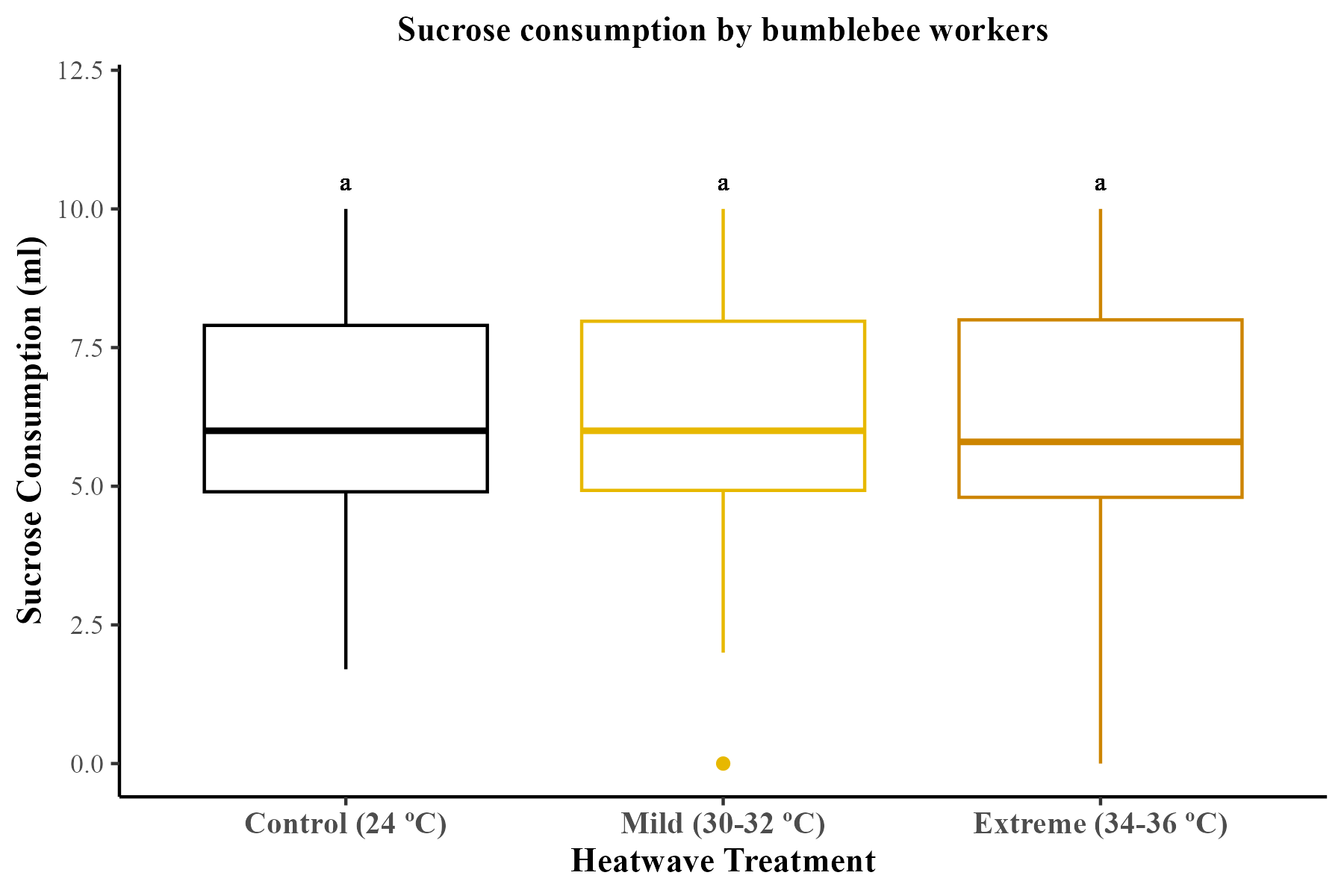

Supplement: Supplemental Information 2 — Nectar consumption (ml) was measured throughout the five-week study period for the 29 bumblebee microcolonies. The boxes represent the interquartile ranges and the horizontal lines within the boxes show the medians. Whiskers extend to 1.5 times the interquartile range from the first and third quartiles, displaying the range of the data. Any data points outside these whiskers are considered outliers and are represented as individual points. [file peerj-12-17135-s002.png]

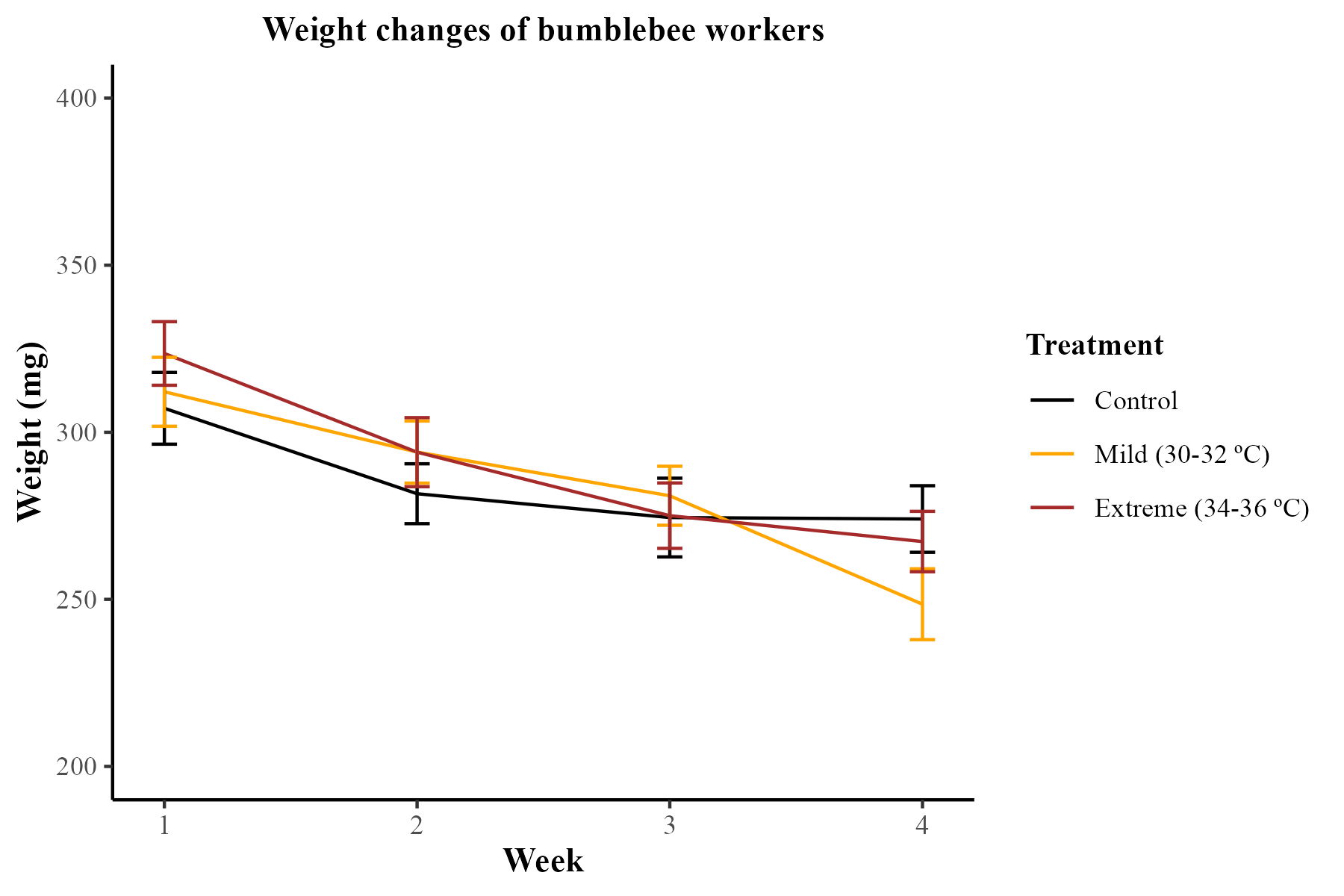

Supplement: Supplemental Information 3 — Lines represent mean values and standard errors (SE) of bumblebee weight for each treatment and week. [file peerj-12-17135-s003.png]

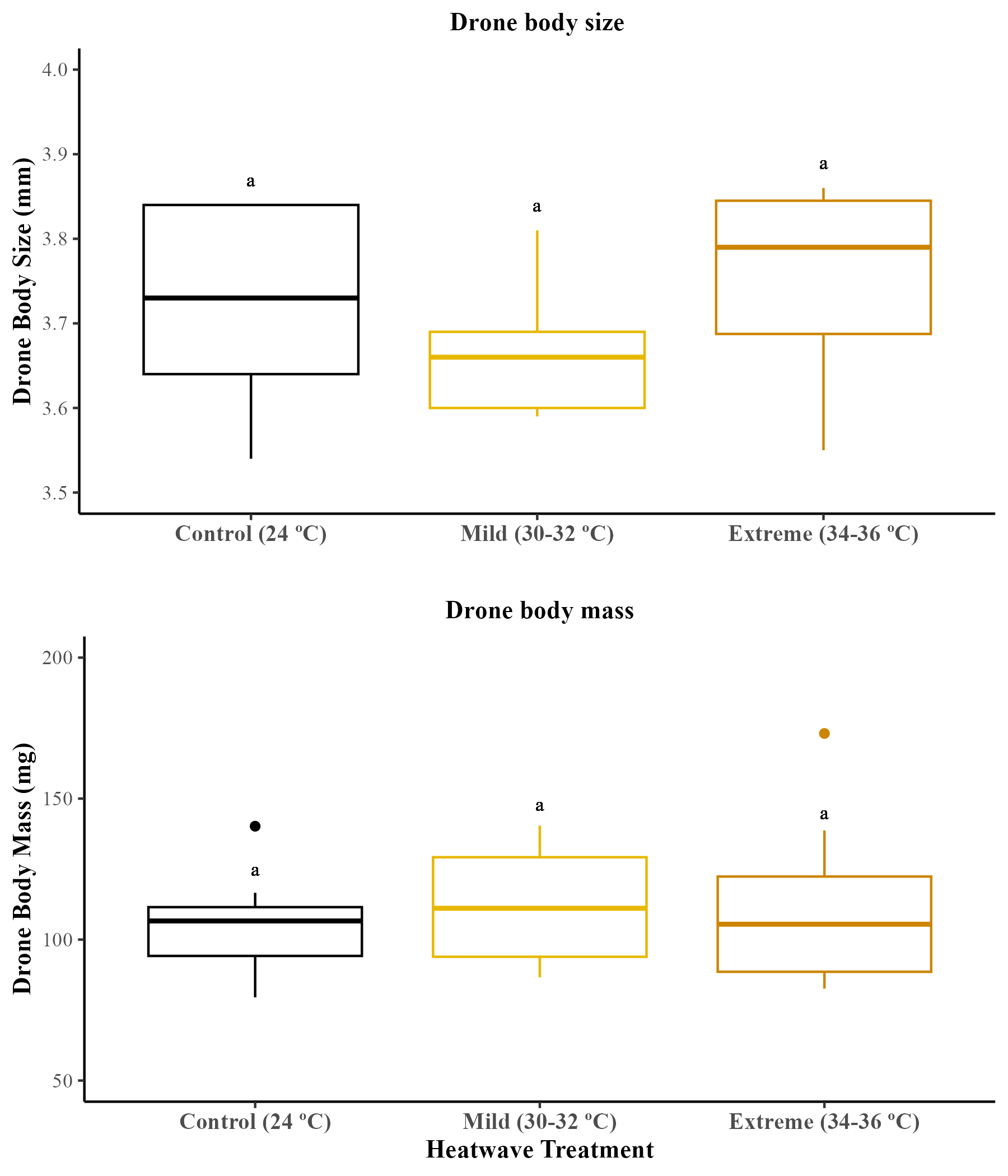

Supplement: Supplemental Information 4 — The body size (Control = 33 bees; mild heatwave = 55 bees; extreme heatwave = 54 bees) and the body mass (Control = 39 bees; mild heatwave = 68 bees; extreme heatwave = 59 bees) of drones across the 29 microcolonies were measured after the five-week study period. The boxes represent the interquartile ranges and the horizontal lines within the boxes show the medians. Whiskers extend to 1.5 times the interquartile range from the first and third quartiles, displaying the range of the data. Any data points outside these whiskers are considered outliers and are represented as individual points. [file peerj-12-17135-s004.png]
